# Dual-Domain Impulse Complexity Index-Guided Projection Iterative-Methods-Based Optimizer-Feature Mode Decomposition (DICI-Guided PIMO-FMD): A Robust Approach for Bearing Fault Diagnosis Under Strong Noise Conditions

**DOI:** 10.3390/s25196174

**Published:** 2025-10-05

**Authors:** Dongning Chen, Qinggui Xian, Chengyu Yao, Ranyang Deng, Tai Yuan

**Affiliations:** 1 School of Mechanical Engineering, Yanshan University, Qinhuangdao 066004, China; 18629448551@163.com (Q.X.); rydeng@stumail.ysu.edu.cn (R.D.);; 2 Hebei Key Laboratory of Industrial Computer Control Engineering, Yanshan University, Qinhuangdao 066004, China; chyyao@ysu.edu.cn

**Keywords:** bearings, low signal-to-noise ratio, dual-domain impulse complexity index, feature mode decomposition, projection-iterative-methods-based optimizer

## Abstract

Bearings are core components in many types of industrial equipment, and their operating environment is often accompanied by strong background noise. This results in a low Signal-to-Noise Ratio (SNR) in the collected vibration signals, making it difficult for traditional methods to extract fault information effectively. Given that bearing failures often manifest as periodic impact signals, a Feature Mode Decomposition (FMD) method has been proposed by researchers which optimizes filter design through correlated kurtosis to enhance the ability to capture fault impact components. However, the decomposition performance of FMD is significantly affected by its parameters (mode number and filter length), and relies on manual settings, resulting in insufficient stability of the results. Therefore, this paper proposes a Dual-domain Impulse Complexity Index (DICI) that combines time-domain impulse characteristics and frequency-domain complexity as an evaluation criterion for FMD parameter optimization. Further, the projection-iterative-methods-based optimizer (PIMO) is adopted to achieve adaptive optimization of parameters. Subsequently, sensitive components are selected based on the maximum Fault Frequency Correlation (FFC) criterion, and their envelope spectra are calculated to recognize bearing fault modes. Simulation and real-signal verification show that the proposed method outperforms several established signal-processing approaches under low SNR conditions.

## 1. Introduction

Bearings play an irreplaceable role in industrial production [1], and accurate monitoring of their operational status is crucial to ensure the safety and continuity of the production process [2]. However, bearings typically operate in extreme conditions of high speed and strong noise, and the collected vibration signals are highly susceptible to interference from environmental noise [3]. Therefore, there is an urgent need to develop a high-precision and highly robust fault diagnosis method suitable for strong noise environments.

Bearing failures typically stem from friction, wear, fatigue, corrosion, and lubrication failure. For instance, Tandon et al. [4] analyzed the root causes of bearing vibration and the corresponding vibration responses associated with different defect types. Given the critical role of bearings in mechanical systems, effective preventive measures must be implemented: moisture intrusion should be prevented in the lubrication system [5]; and an appropriate lubricant must be selected [6].

Bearing vibration signals are an important subject for researchers studying bearing faults. For instance, Tang et al. [7] proposed a lifelong diagnostic method using inverted transformers with a learnable pruning mechanism based on bearing vibration signals; Nie et al. [8] developed a novel diagnostic approach that integrates image enhancement and improved convolutional neural networks by utilizing vibration signals.

Vibration signals of bearing faults are typically characterized by periodic transient impacts caused by localized defects [9]. Spectrum analysis is an effective fault diagnosis method for bearings. Effectively extracting this pure impact under strong background noise using spectrum analysis remains the core challenge in fault diagnosis. Existing research generally adopts a two-step strategy of “decomposition first, reconstruction later” to eliminate the various interferences: first, signal decomposition technology is used to serialize the original observations into several sub-modes, and then components highly correlated with the impact components are selected based on specific criteria to complete reconstruction. The methods based on the above strategy include Singular Geometric Mode Decomposition (SGMD) [10], Empirical Mode Decomposition (EMD) [11], Variational Mode Decomposition (VMD) [12], Complete Ensemble Empirical Mode Decomposition with Adaptive Noise (CEEMDAN) [13], Wavelet Packet Decomposition (WPD) [14], Morphological Filtering (MF) [15], and Fast Iterative Filtering (FIF) [16], etc.

In the reconstruction process, components can be selected mainly by obeying two kinds of criteria: the first is the complexity screening criterion, which removes components with significantly higher entropy values (exceeding the threshold) to retain high regularity components. For example, Pan et al. [17] used Improved Complete Ensemble Empirical Mode Decomposition with Adaptive Noise to obtain modal components, calculated the permutation entropy of each component, and then used the mean permutation entropy as a threshold to screen modal components. The second criterion is the correlation coefficient criterion: filtering components based on the correlation coefficient between the component and the original signal, and retaining the components with higher correlation. For example, Jiang et al. [18] first obtained modal components via EEMD, then reconstructed the signal by selecting significant components based on the correlation coefficient.

The performance of signal decomposition techniques is highly dependent on preset hyperparameters. An example is Variational Mode Decomposition (VMD), which is the selection of the number *K* of decomposition modes. To overcome the uncertainty caused by empirical parameter setting, scholars built a fitness function based on certain criteria and then used an optimization algorithm to achieve optimal hyperparameters. Li et al. [19] constructed an optimization model that uses maximum envelope kurtosis as the criterion to optimize VMD parameters; Wang et al. [20] proposed the use of relevant waveform indices as fitness functions and utilized the Archimedes optimization algorithm to dynamically adjust VMD hyperparameters. Wavelet thresholding denoising [21] is a good denoising method, but its performance is affected by multiple parameters such as threshold. When the threshold is too small, it can lead to incomplete denoising; When the threshold is too high, it causes signal distortion. Due to this, Hu et al. [22] used a genetic algorithm to optimize the parameters of wavelet threshold denoising. The above work indicates that constructing a fitness function closely coupled with diagnostic objectives can effectively solve the problem of difficult parameter adaptation in decomposition models.

The above method can effectively separate signal components, but its decomposition mechanism doesn’t specifically aim to extract periodic impulse features. Therefore, based on the idea of deconvolution, a Feature Mode Decomposition (FMD) [23] method is proposed: accurate extraction of periodic impulse signals from the original signal can be achieved by constructing an initial filter and designing a cyclically computable kurtosis index to quantify the transient impulse and periodicity of pulses simultaneously. Compared to existing methods, FMD does not require prior knowledge of the fault cycle and exhibits better noise robustness. However, traditional FMD still relies on manually preset key parameters and lacks parameter adaptability; In addition, the cyclic kurtosis index lacks robustness in strong noise environments. To overcome the above limitations, two innovations are provided in this paper:

(1) Propose a Dual-domain Impulse Complexity Index (DICI) that can achieve maximum values for periodic impulse signals even under low SNR conditions compared to other indicators. Propose an improved cycle estimation method for the fault signal. Give a Fault Frequency Correlation (FFC) method to select the mode component that contains the most fault information.
(2) Propose to use the PIMO algorithm to optimize the parameters of FMD, overcoming the difficulty of parameter selection.

## 2. Proposed Method

In this section, spectrum analysis is used to diagnose the bearing fault, and a series of new methods are proposed to improve the diagnostic capacity under strong noise conditions.

### 2.1. A New Method of Period Estimation

The decomposition performance of FMD largely depends on the quality of the filter, and the update of the filter depends on the period of the signal. Therefore, an accurate period estimation formula is crucial for the final decomposition result.

Traditional period estimation usually relies on the local maximum of the time-domain autocorrelation function for judgment, but its positioning accuracy is significantly limited under strong noise and multi-harmonic interference conditions. Therefore, this paper proposes a new strategy for period estimation based on the envelope spectrum (*ES*). Given the fault characteristic frequency *f_FCF_*, theoretically, equidistant harmonic peaks will appear at integer multiples, and the minimum positive period of this harmonic structure corresponds to the fault period. To enhance the fundamental frequency component and suppress false peaks, the following weighting function is constructed:

(1) $$\omega_{m}=a^{m-1},\;m=1,\ldots ,M$$

where *M* is the number of harmonics obtained, and it is taken as 5 and in this paper, *a* is a constant with a value of 0.8.

Calculate the periodicity of the signal according to the following equation

(2) $$R_{ES}(f)={\left(\prod_{m=1}^{M}\omega_{m}ES(mf)\right)}^{\frac{1}{M}},f=1,2,3,\ldots ,\frac{f_{s}}{2}$$

where *f_s_* is the sampling frequency, and *ES* is the envelope spectrum.

(3) $$f_{FCF}=\arg\max\{R_{ES}(f)\},f=1,2,3,\ldots ,\frac{f_{s}}{2}$$

where *f_FCF_* is the fault characteristic frequency.

The fault period *T* (*T* represents the number of points corresponding to one cycle) can be obtained as follows:

(4) $$T=\mathrm{round}\left(\frac{f_{s}}{f_{FCF}}\right)$$

### 2.2. Dual-Domain Impulse Complexity Index (DICI)

At present, many studies have focused on the fault diagnosis of bearings, and many calculation indicators have been proposed to measure the fault information contained in bearing signals. Cheng et al. [24] proposed a bearing fault diagnosis method based on a spectral-correlation-improved envelope spectrum; Wang et al. [25] used the L1/L2 norm of the spectrum as a measure of bearing fault signals; Wang et al. [26] constructed a framework for diagnosing bearing failures based on kurtosis, negative entropy, Gini index, and smoothness index; Miao et al. [27] proposed a parameter index of Harmonic Noise Ratio (HNR) to measure the fault characteristics of bearings to solve the problem of the unclearness of early failures; Cheng et al. [28] uses Cyclic Kurtosis (CK) as the basis for selecting the eigenmode function after signal decomposition, to amplify the periodic impact components in the bearing signal. The above indicators use the time or frequency domain to quantify the impulse and periodicity of periodic shocks, but in low SNR situations, the indicators cannot effectively screen out the periodic impulse signal. Therefore, this paper proposes a new calculation metric.

**Figure 1:**
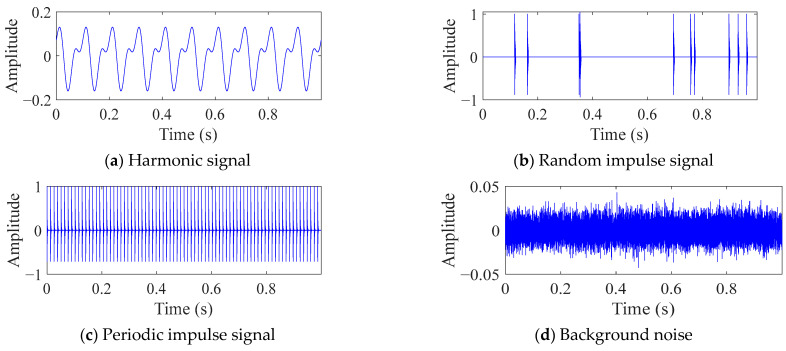
Time domain diagrams of a signal composed of four types of signals.

**Figure 2:**
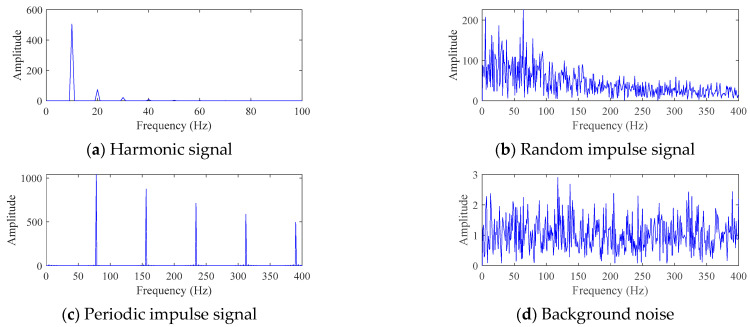
The envelope spectrum of a signal composed of four types of signals.

The machinery fault signal can usually be understood as a composition of four signals: harmonic signal, random impulse signal, periodic impulse signal, and background noise (also known as white noise). Draw the time-domain diagrams of the four signals using the data from Section 3.1, as shown in Figure 1. The amplitude of white noise is 0.01. The corresponding envelope spectrum is shown in Figure 2.

For the four signals in Figure 1, the fault signal is mainly reflected as the periodic impulse signal. Therefore, designing a calculation metric that maximizes the periodic pulse signals among the four signals can serve as the objective function for optimizing FMD, thereby obtaining a larger proportion of periodic pulse signals in the results. Although noise can theoretically be removed through filtering out the four types of signals, data collected during actual industrial production will be strongly affected by background noise interference, and traditional filtering methods have certain difficulties in extracting periodic pulse signals. Therefore, this paper proposes a new indicator named the dual-domain impulse complexity index (DICI), which can still have good recognition ability of fault signal in low SNR environments.

DICI considers the complexity and impulse characteristics of signals from both frequency and time domains.

For the given signal *x*(*n*), *n* = 1, 2, ⋯, *N*,

(5) $$\left\{\begin{array}{c} R_{SE(\tau )}=\sum_{n=1}^{N}SE(n)SE(n+\tau )\\ SE(x(t))={\left|x\right|}^{2}={\left|x+j•Hilbert(x(t))\right|}^{2} \end{array}\right.$$

(6) $$Hilbert(x(t))=\frac{1}{\pi }\int_{-\infty }^{\infty }\frac{x(t)}{t-\tau }d\tau$$

where *SE*(*x*) represents the squared envelope signal of *x*(*n*), *R_SE_*(*x*) denotes the autocorrelation function of *SE*(*x*), *τ* is the time delay, and *Hilbert*(.) represents the Hilbert transformation operator. As a linear operator, Hilbert transformation operator converts a real-valued signal into a complex signal by applying a -90-degree phase shift to all frequency components.

Then we present the novel indicator as follows:

(7) $$DICI(x,T)=\left(\frac{\sum_{i=1}^{M}R_{SE(x)}(iT)}{R_{SE(x)}(0)}\right)\times \left(1-\frac{e^{\mathrm{mean}(\ln(ES))}}{\mathrm{mean}(ES)}\right)$$

where *ES* represents the envelope spectrum of the signal, *iT* (*i* = 1, 2, ⋯, *M*) is the time delay coefficient, and *M* in this study is suggested as 3 [29].

DICI divides the index into two parts based on multiplication symbols. The first part mainly quantifies the impulse characteristics in the time domain. While the second part mainly quantifies the complexity of the frequency domain by calculating the flatness of the signal ES. To this end, this indicator combines time and frequency domains, allowing periodic impulse signals to reach their maximum values.

Subsequently, we select some existing indicators for comparison, such as Envelope Harmonic-to-Noise Ratio (EHNR) [30], Signal Cycle Kurtosis-to-Noise Ratio (SCKNR) [29], Sparsity-Complexity Integration Measure (SCIM) [31], and Shannon Entropy (SE) [32].

(8) $$CK(x,T)=\frac{\sum_{i=1}^{M}R_{SE(x)}(iT)}{{\left[R_{SE(x)}(0)\right]}^{2}}$$

(9) $$EHNR=\frac{R_{SE(x)}(\tau_{\max})}{R_{SE(x)}(0)-R_{SE(x)}(\tau_{\max})}$$

(10) $$HNR=\frac{R_{SE(x)}(T)}{R_{SE(x)}(0)-R_{SE(x)}(T)}$$

(11) $$SCIM(x,T)=-\sum_{i=1}^{M}\left(\frac{R_{SE(x)}(iT)}{\sum_{i=1}^{M}R_{SE(x)}(iT)}\log_{2}\frac{R_{SE(x)}(iT)}{\sum_{i=1}^{M}R_{SE(x)}(iT)}\right)$$

(12) $$SCKNR(x,T)=10\log_{10}(\frac{\sum_{i=1}^{M}R_{SE(x)}(iT)}{\sum_{\tau =1}^{N-1}R_{SE(x)}(\tau )-\sum_{i=1}^{M}R_{SE(x)}(iT)})$$

This paper uses Equation (13) to add white noise with different SNRs to the four signals mentioned above for testing.

(13) $$SNR=10\log_{10}(\frac{Psignal}{Pnoise})=10\log_{10}(\frac{\sum_{n=1}^{N}SE(x_{s})}{\sum_{n=1}^{N}SE(x)-\sum_{n=1}^{N}SE(x_{s})})$$

**Figure 3:**
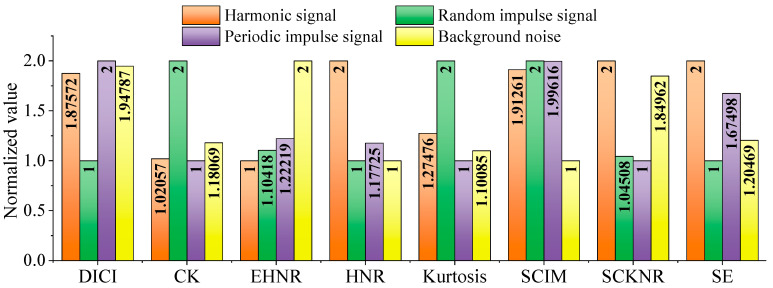
Comparison chart between DICI and other indicators.

Using the above indicators, calculate the values of the four types of signals separately, and normalize the results to obtain Figure 3. The SNR of white noise is set as −15 dB. At the same time, to avoid the problem of zero values after normalizing data of the same type, the results are uniformly incremented by 1.

As shown in Figure 3, under extremely low SNR ratio conditions, the proposed index DICI of this paper can still maximize the amplitude of periodic impulse components, while other indexes struggle to achieve this effect. In Figure 3, other indicators except DICI are constructed only in the time domain, and after being affected by strong white noise, the time domain signal often appears highly chaotic, leading to a significant decrease in the performance of time domain indicators. This proposed index DICI utilizes both time-domain and frequency-domain information, effectively suppressing noise interference and exhibiting stronger robustness.

### 2.3. Fault Frequency Correlation (FFC)

The fault characteristics of rotating machinery are usually presented as periodic transient effects, and their spectra exhibit significant peaks at the fault characteristic frequencies and their harmonics. To effectively extract the most valuable diagnostic components, it is necessary to construct a modal screening mechanism oriented towards fault impulse saliency. So this paper proposes a fault frequency correlation (*FFC*) to quantify the degree of matching between each mode and the fault characteristics.

The specific expression of fault frequency correlation is as follows

(14) $$FFC=\frac{R_{SE(x)}(T_{fault})}{R_{SE(x)}(0)}$$

where *T_faul_*_t_ is the actual cycle of the fault signal.

Specifically, we perform time-domain analysis on each sub-mode, calculate the correlation proportion when the delay is the actual cycle and the delay time is 0, and use the mode corresponding to the maximum value of FFC as the optimal component for subsequent fault diagnosis.

### 2.4. PIMO-FMD

Feature mode decomposition is a novel signal decomposition method that is mainly used for feature extraction of machinery fault signals. In this section, a FMD optimized by PIMO is proposed to avoid the improper manual selection of hyperparameters used in the original FMD.

#### 2.4.1. The Original Feature Mode Decomposition

As mentioned above, a machinery fault often consists of four types of signals: harmonic signals, random impact signals, periodic impact signals, and background noise. Exacting periodic impulse signals is very important as it is the fault feature signal. In the method of FMD, different modes are decomposed by designing adaptive finite impulse response (FIR) filters. Then, iterative thinking and the correlated kurtosis methods are employed to capture filtered signals with good periodicity and impulse characteristics.

The specific step of FMD is as follows:

Step 1: Input the relevant parameters of the original signal *x* and FMD.
Step 2: Initialize the filter, with current iteration count *i* = 1;
Step 3: Perform filtering operation according to $u_{m}^{i}=x*f_{m}^{i}$, where $f_{m}^{i}$ is the *m*-th filter at the *i*-th iteration, $u_{m}^{i}$ is the *m*-th filtered signal at the *i*-th iteration, and * represents the convolution operation;
Step 4: Update the parameters of the filter using the filtered signal. Determine whether the maximum number of iterations has been reached. If it reaches, proceed to step 5; otherwise, return to Step 3;
Step 5: Calculate the correlation coefficient between adjacent components, construct a correlation coefficient matrix, select the group with the highest correlation coefficient, and screen the signal component with a larger correlated kurtosis. Finally, define *M* = *M* − 1;
Step 6: Repeat the above steps until *M* signal components are output.

#### 2.4.2. Parameter Optimization of FMD

**Figure 4:**
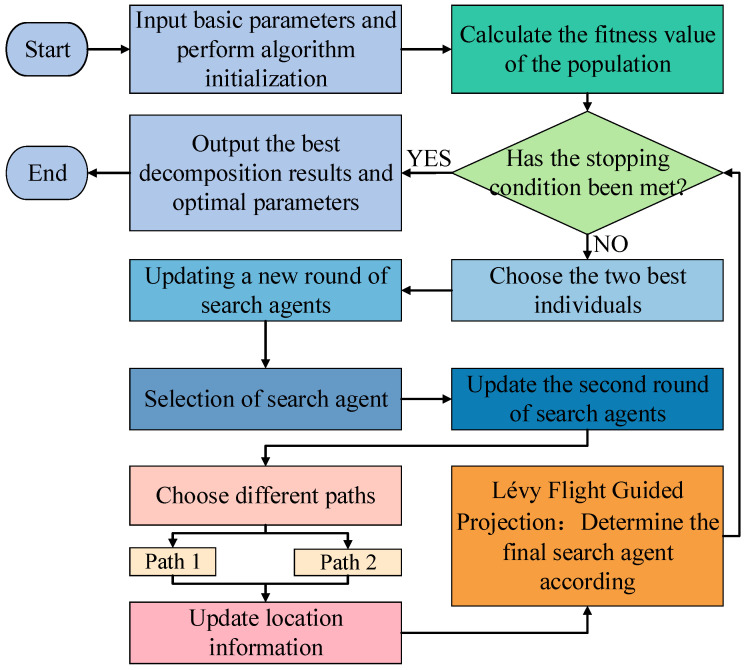
Process diagram of PIMO-FMD.

FMD can adaptively decompose complex signals into several modes and has a certain robustness to noise, but its decomposition performance is affected by two hyperparameters: the number of modes *K* and the filter length *L*. If *K* is too small, incomplete decomposition will occur, and if *K* is too large, it will cause excessive decomposition; an *L* that is too short may cause information loss, while an *L* that is too large can significantly increase computational complexity. Therefore, it is necessary to construct a fitness function with physical significance and use optimization algorithms to automatically optimize *K* and *L* at once, achieving true adaptive decomposition. This paper introduces an optimizer to select the most suitable *K* and *L*, and the optimizer is a projection-iterative-methods-based optimizer (PIMO) [33]. PIMO is inspired by the principle of geometric projection and constructs four new operators. In PIMO, for the first time, the Kaczmarz method is organically integrated with stochastic gradient descent to enhance global search capability while effectively avoiding becoming stuck in local optima. The corresponding PIMO-FMD process is shown in Figure 4.

### 2.5. Fault Diagnosis of DICI Guided PIMO-FMD

The decomposition performance of the newly proposed feature mode decomposition (FMD) is affected by the period estimation strategy and two hyperparameters. Therefore, this paper proposes a DICI Guided PIMO-FMD algorithm to solve the above problems, and the steps of fault diagnosis are as follows:

Step 1: Input the collected time series signal. Set the basic parameters of PIMO.
Step 2: Set the following equation as the objective function, which is the DICI proposed in this paper, and optimize the parameters of FMD using PIMO.
  (15) $$\underset{p_{i}=(K,L)}{\arg\max}\{DICI_{p_{i}}\},\;\mathrm{subject}\;\mathrm{to}\;K\in \;[2,7]\;\mathrm{and}\;L\in [2,230]$$
  where *p_i_* is the position information of the *i*-th individual in the population, and *DICIp_i_* is its corresponding fitness value.
Step 3: Reconstruct the signal using FFC and calculate its envelope spectrum, and then complete the diagnosis based on the obtained fault frequency.

**Figure 5:**
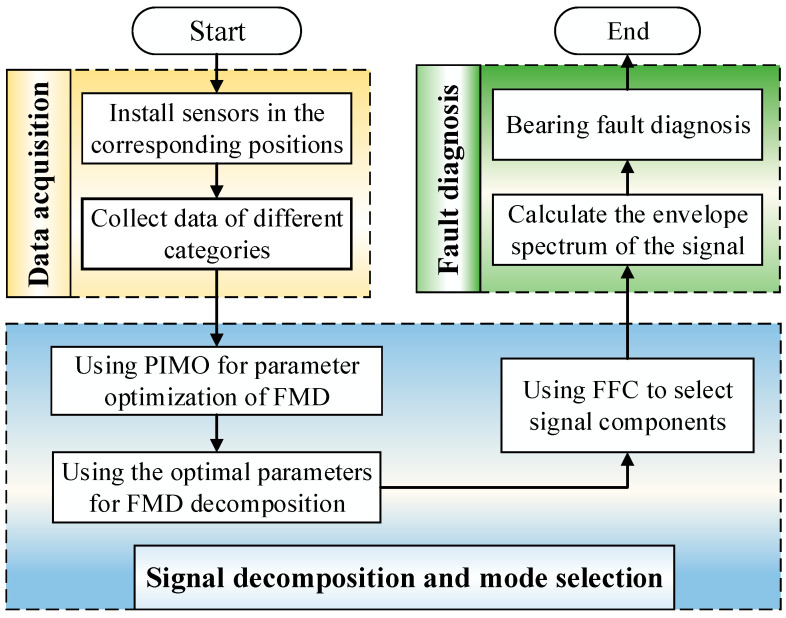
Diagnostic process based on DICI Guided PIMO-FMD.

The corresponding diagnostic flowchart is shown in Figure 5.

## 3. Verification of Simulated Bearing Signals

In this section, a bearing vibration signal model is used to generate simulated signals containing bearing outer ring faults [34] to demonstrate the effectiveness of the proposed method DICI Guided PIMO-FMD.

### 3.1. Signal Construction

In actual working conditions, the measured signal is usually not a pure periodic impulse signal, but a mixed signal containing random pulses, harmonic interference, and Gaussian white noise. The bearing vibration signal model is as follows.

(16) $$x(t)=\sum_{i}a_{i}h_{1}(t-iT_{A})+\sum_{j}b_{j}h_{2}(t-jT_{B})+\sum_{k}c_{k}\sin(2\pi f_{k}t+\theta_{k})+n(t)$$

In Equation (16), the bearing vibration signal consists of four components. The first component originates from the periodic impact generated by the rolling element each time it passes through the fault point; The second component corresponds to random impacts caused by accidental collisions or other external disturbances. The structural resonance induced by these two types of impacts can be described uniformly using the time-domain response function *h_i_*(*t*), and its specific form is given in the following text.

(17) $$h_{i}(t)=\left\{\begin{array}{c} e^{-\beta_{i}t}\cos(2\pi f_{ni}t)\;t>0\\ 0\;\;\;\;\;\mathrm{otherwise} \end{array}\right.$$

where *β_i_* represents the impulse damping coefficient, and *f_ni_* denotes the resonance frequency.

The third term represents harmonic interference generated by shaft rotation, and the fourth term is Gaussian noise, simulating real engineering conditions. The specific parameters are shown in Table 1. The sampling frequency for collecting analog signals is 16,384 Hz, and the sampling time is 1 s [34].

**Figure 6:**
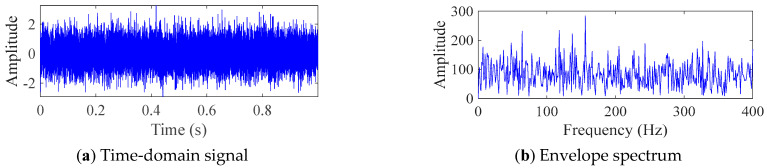
Time domain diagram and envelope spectrum diagram of simulated bearing signals.

Figure 6 shows the time domain diagram and envelope spectrum diagram of simulated bearing signals. As shown in Figure 6, due to the influence of white noise with a SNR of −13, a periodic impulse signal cannot be observed in the time domain, and the corresponding fault frequency cannot be observed in the frequency domain. This indicates that the simulated fault signal is consistent with the signals collected in actual production activities. In the PIMO algorithm, the population size is 30, the maximum number of iterations is 50, the upper limit of the search is *ub* = [7, 230], and the lower limit of the search is *lb* = [2, 2]. The maximum number of iterations in FMD is set to 30.

The simulations in this study were performed on a laptop computer using MATLAB R2020a. The specifications of the laptop are as follows: Operating System: Windows 11; CPU: Intel Core i9-13900HX; GPU: NVIDIA GeForce RTX 4060. Using the method described in this article, the envelope spectrum can be obtained in just 0.22 s after determining the optimal parameters.

### 3.2. Comparison of Fault Diagnosis Methods

To validate the effectiveness of the proposed DICI Guided PIMO-FMD in this paper, multiple classic methods were selected for comparison, as shown in Table 2. DICI Guided PIMO-TT-FMD is the same method as DICI Guided PIMO-FMD, but it uses the origin period estimation method to verify the effectiveness of the proposed period estimation method.

**Figure 7:**
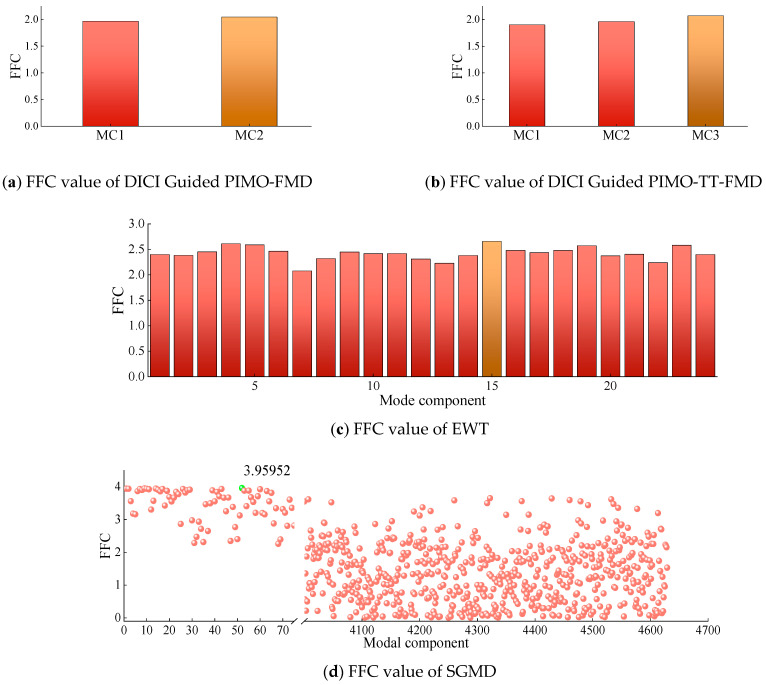
FFC value of modal components for simulating data.

The method of selecting modal components using FFC is adopted in DICI Guided PIMO-FMD, DICI Guided PIMO-TT-FMD, EWT, and SGMD, and the corresponding FFC for each modal component (MC) is shown in Figure 7.

**Figure 8:**
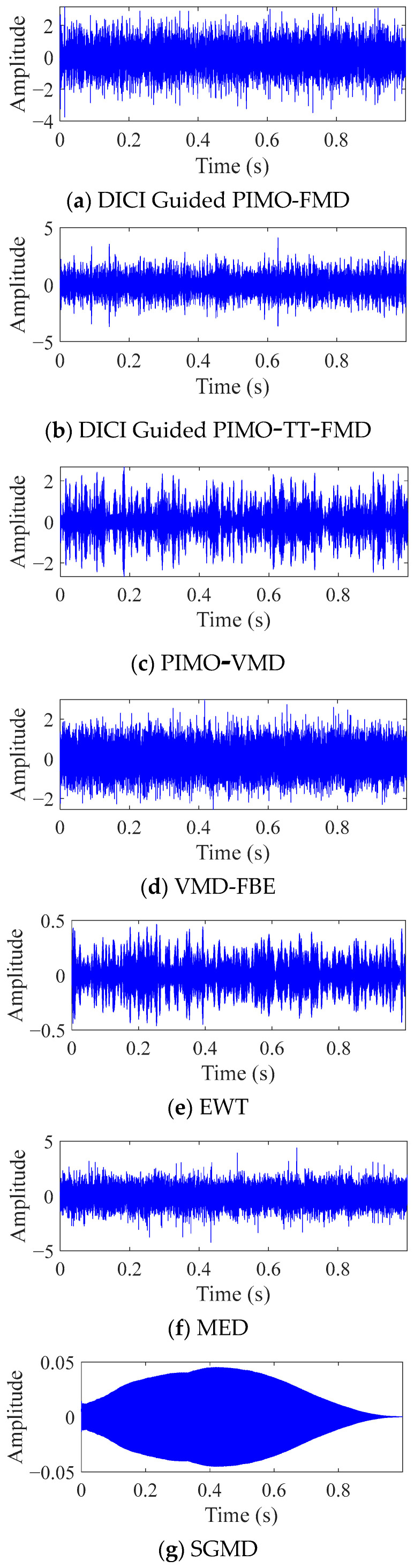
Time domain graph of simulated data processed by different methods.

**Figure 9:**
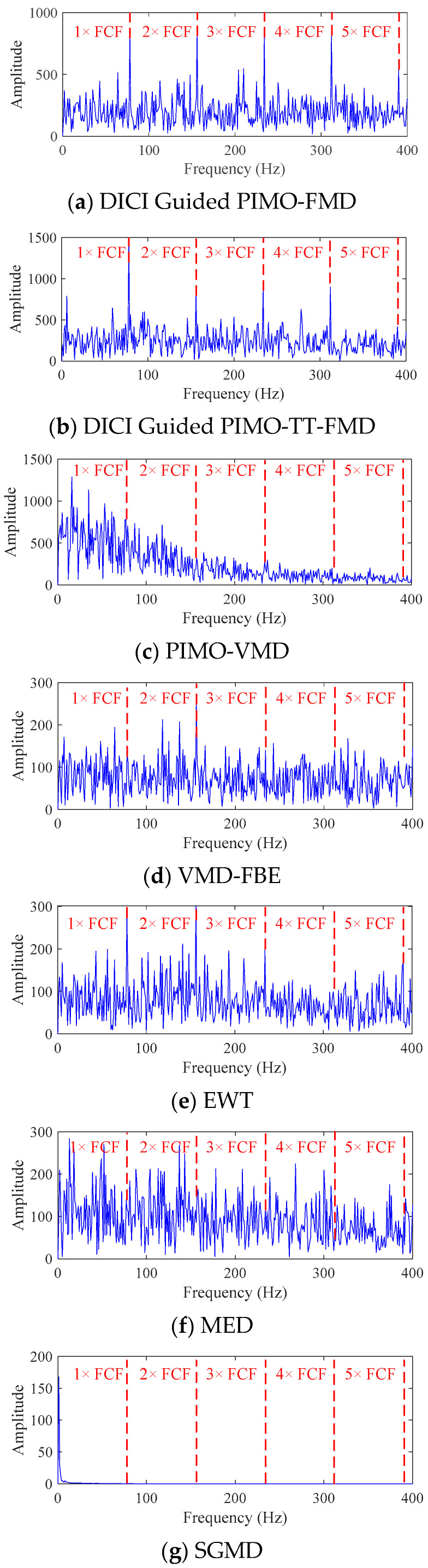
Envelope spectra of simulated data graph processed by different methods.

As shown in Figure 7, the best results of DICI Guided PIMO-FMD and DICI Guided PIMO-TT-FMD are *K* = 2 and *L* = 25, respectively, and *K* = 3 and *L* = 41, respectively. By calculating the FFC values of each modal component, it can be concluded that DICI Guided PIMO-FMD takes the second modal component and DICI Guided PIMO-TT-FMD takes the third modal component; EWT takes the 15th modal component, and SGMD takes the 52nd modal component. Figure 8 and Figure 9 show the time-domain and frequency-domain graphs of the signals processed by the seven different methods of Table 2.

For DICI Guided PIMO-FMD, the time-domain graph Figure 8a shows that the signal exhibits significant periodic impulse characteristics; The envelope spectrum of Figure 9a forms sharp and prominent spectral peaks at the characteristic frequency of bearing faults, which can achieve precise positioning of fault characteristic frequencies.

For DICI Guided PIMO-TT-FMD, which does not using the proposed the period estimation method, the time-domain graph of Figure 8b exhibits significant periodic impulse characteristics; However, in the envelope spectrum of Figure 9b, only the first four fault characteristic frequencies show obvious spectral peaks, the peak at the fifth fault frequency is missing, and there is a significant interference component of 278 Hz near the third and fourth fault frequencies. The above results indicate that the improved period estimation method proposed in this paper has better performance in suppressing interference and highlighting target harmonics.

To further validate the universality of the proposed method, we used VMD [38] as the comparative decomposition model and replaced the grasshopper optimization algorithm (GOA) with PIMO, while keeping the objective function of the weighted kurtosis index. As shown in Figure 8c, the time-domain signal of PIMO-VMD does not exhibit significant periodic impulse characteristics, and no clear spectral peaks related to faults are detected in the envelope spectrum in Figure 9c.

For VMD-FBE, according to the processed time-domain signal of Figure 8d, there is no obvious periodicity, and effective fault information cannot be extracted from the corresponding envelope spectrum of Figure 9d.

For EWT, the periodic characteristics of its time-domain waveform in Figure 8e are not significant; The corresponding envelope spectrum of Figure 9e only shows significant peaks at 1 × FCF and 2 × FCF, while there are several small amplitude spurious peaks in the 2 × FCF-3 × FCF and 4 × FCF-5 × FCF intervals, which can easily interfere with subsequent fault diagnosis; In addition, no significant peak can be identified at 4 × FCF.

Minimum Entropy Deconvolution (MED), as a classic method for enhancing bearing fault features, is also introduced for comparative verification. This paper sets the length of the MED filter to 35 and the number of iterations to 100. As shown in Figure 8f and Figure 9f, the time-domain waveform after MED denoising does not exhibit significant periodic impulse characteristics; No clear spectral peaks corresponding to the fault were detected in its envelope spectrum.

As shown in Figure 8g and Figure 9g, the signal processed by SGMD did not exhibit significant periodic impact characteristics in the time domain, and no clear spectral peaks corresponding to bearing faults were detected in the frequency domain.

Based on the above results, it can be seen that our proposed DICI Guided PIMO-FMD has superiority compared to other common methods in fault recognition under low SNR conditions.

### 3.3. Validation of Different Indicators

**Figure 10:**
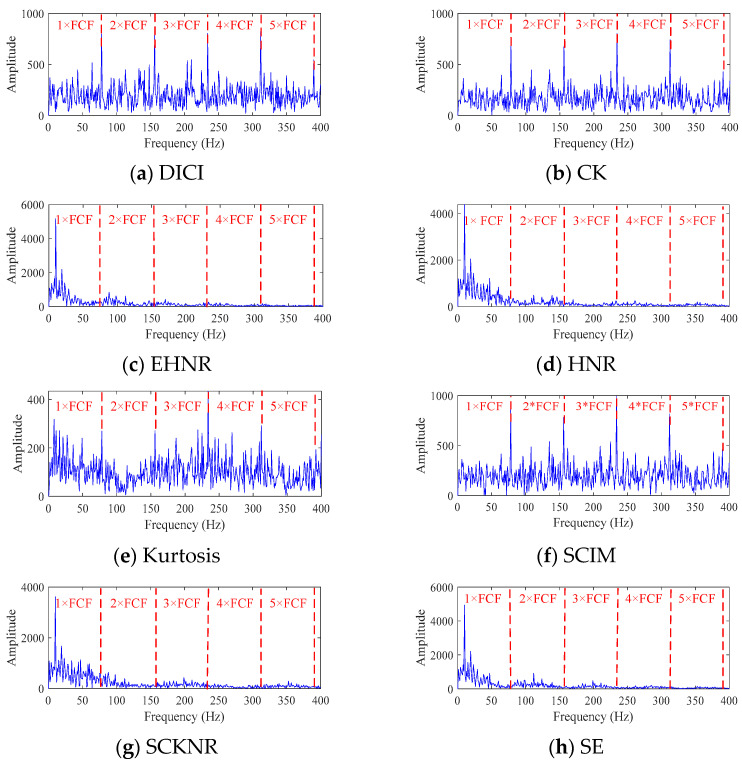
Envelope spectra decomposed by different indicators.

To further validate the effectiveness of the proposed indicator, DICI, PIMO-FMD was used as the signal decomposition method, and the remaining indicators in Section 2.2 were used as the decomposition objective function, with the FFC method used to select the components. The envelope spectra of different methods are then plotted, with details shown in Figure 10.

As shown in the Figure 10, when EHNR, HNR, SCHNR and SE are used as objective functions, the decomposition fails to capture any fault-characteristic frequencies due to the introduction of low-SNR components. In contrast, adopting CK, Kurtosis and SCIM as objectives allows the first four fault frequencies to be detected, yet their amplitudes at 5 × FCF are all lower than those obtained with the proposed DICI indicator. Therefore, DICI delivers the best fault-detection performance.

## 4. Experiment

### 4.1. Data Collection

This paper selected the bearing acceleration life experiment of XJTU-SY [39] for verification. The last dataset of the outer ring fault is selected. The sampling frequency of this data is 25.6 kHz, and the rotation frequency of the bearing is 37.5 Hz. Due to the difference between the data acquisition of the experimental bench and the strong noise environment in real production, this paper adds white noise with a SNR of -7 to simulate real life and production. The specific experimental bearing parameters are shown in Table 3. After calculation using these parameters, the fault characteristic frequency of the bearing is 115.6 Hz.

**Figure 11:**
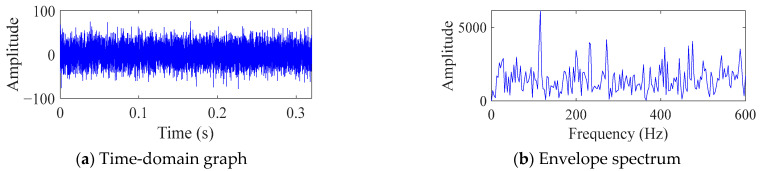
Collecting signals.

The corresponding time-domain signal and frequency-domain graph are shown in Figure 11. It can be seen that there is no obvious periodic impulse in the signal, and only 1 time the FCF can be observed in Figure 11b, and no other fault frequencies can be observed.

### 4.2. Comparison of Fault Diagnosis Results

**Figure 12:**
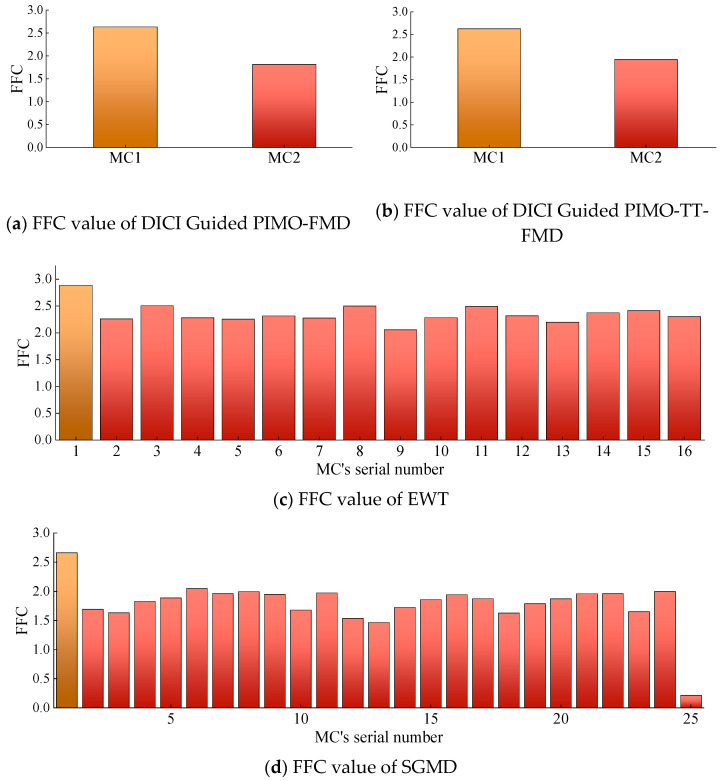
FFC value of modal components of actual data.

The method in Table 2 was chosen for testing in this experiment. The FMD optimized by PIMO has upper and lower limits of [2, 2] and [2, 240]. All other parameters remain unchanged. For the FFC selection method, the FFC values of the modal components corresponding to DICI Guided PIMO-FMD, DICI Guided PIMO-TT-FMD, EWT, and SGMD are shown in Figure 12.

As shown in Figure 12, it can be seen that all the methods for selecting modal components using FFC mentioned above take the first modal component since it is the biggest.

**Figure 13:**
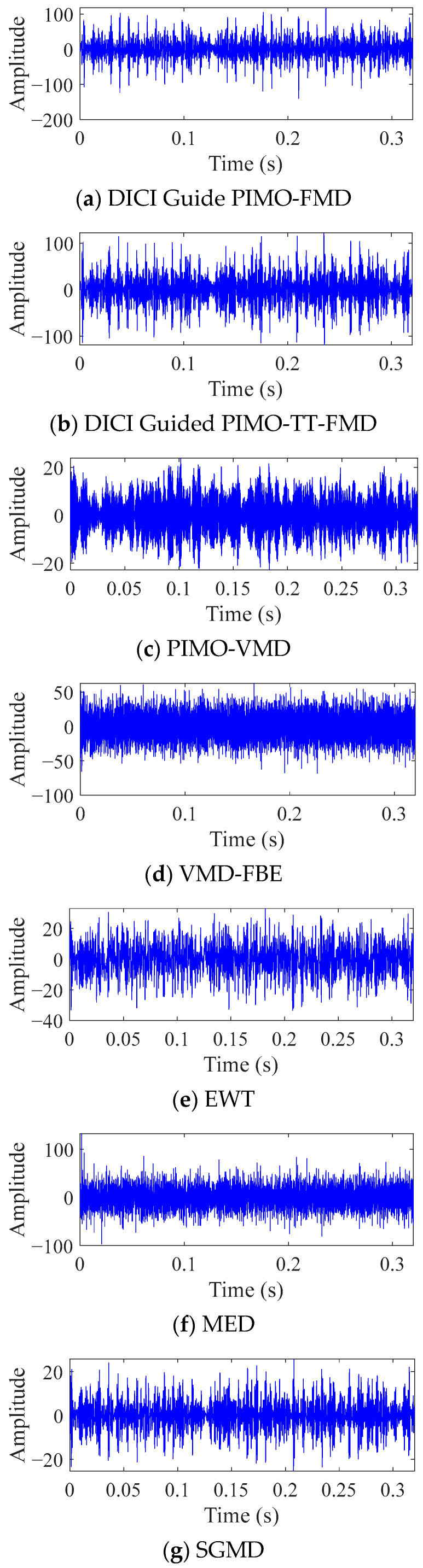
Time domain graph of actual data processed by different methods.

**Figure 14:**
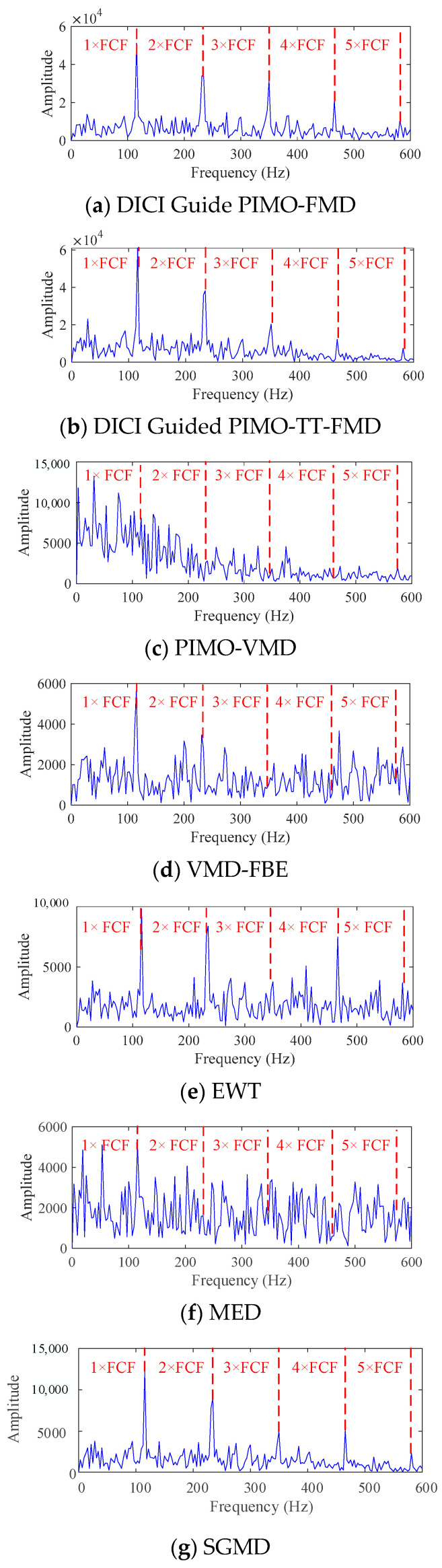
Envelope spectra graph of actual data processed by different methods.

The time-domain and envelope spectra graph corresponding to the processed signal by the seven methods are shown in Figure 13 and Figure 14.

From (a), (b), (g) in Figure 13 and Figure 14, for DICI Guided PIMO-FMD, the optimal mode number and filter length are 2 and 144, respectively; The optimal parameters corresponding to DICI Guided PIMO-TT-FMD are 2 and 63. According to the experimental results, DICI Guided PIMO-FMD, DICI Guided PIMO-TT-FMD, and SGMD can all successfully identify the fault characteristic frequency. However, in terms of spectral resolution, the spectral lines obtained by PIMO-FMD have significant and clear peaks at FCF and its harmonics; In contrast, the relative amplitudes at the 3rd and 4th harmonics in DICI Guided PIMO-TT-FMD are lower than those in DICI Guided PIMO-FMD, and SGMD also shows a similar trend. Overall, DICI Guided PIMO-FMD performed the best in comprehensive fault feature extraction.

As shown in (c)–(f) in Figure 13 and Figure 14, all four methods can only partially restore the fault characteristic frequency. Specifically, VMD-FBE and MED can only identify 1 time the fault characteristic frequency, and the other harmonics cannot be effectively extracted; EWT could detect fault characteristic frequencies one, two, and four times, but there were still missing values. Effective fault information could not be extracted by PIMO-VMD.

In summary, the DICI index proposed in this paper has significant advantages in extracting periodic shock components, and the improved cycle estimation formula also shows higher estimation accuracy compared with the original cycle estimation method. Among all the compared methods, DICI Guided PIMO-FMD exhibits the most outstanding fault feature extraction performance.

## 5. Conclusions

This paper proposes a bearing fault diagnosis method named DICI Guided PIMO-FMD, which is suitable for low SNR environments. Low SNR white noise is injected into bearing simulation signals and laboratory measurement data to reproduce extreme working conditions in actual industrial sites. This paper draws the following conclusions:

(1) This paper proposes DICI, which is significantly superior to existing similar indicators at low SNRs, to ensure that periodic impulse components always achieve maximum response in four typical signals. At the same time, this paper proposes a new signal period estimation method to address the problem of inaccurate period estimation in the original algorithm, and the comparison result of the experiment between DICI Guide PIMO-FMD and DICI Guided PIMO-TT-FMD shows that the new method performs better.

(2) This study proposes a parameter optimization feature mode decomposition (FMD) method based on projection-iterative-methods-based optimizer (PIMO), which automatically searches for the optimal combination of parameters of FMD through the DICI ratio of the signal. This method combines the advantages of PIMO and FMD, and realizes adaptive signal decomposition without prior knowledge and with strong robustness to noise interference. The modal component containing the most fault information is selected using fault frequency correlation. Its diagnostic performance is superior to existing mainstream diagnostic methods.

Future research will extend the above methods to the diagnosis of bearing composite faults and early bearing faults, and explore their applications in other machinery such as pumps and gears.

## Figures and Tables

**Table 1:** Parameters of bearing simulation signal.

Signal	Periodic Pulse			Random Impulse		Harmonic Interference					
Parameter	*β*_1_	*f_n_*_1_	*T*_A_	*β*_2_	*f_n_*_2_	*f*_1_	*θ*_1_	*c*_1_	*f_2_*	*θ*_2_	*c*_2_
Value	1300	6000	1/78	500	2000	10	π/2	0.105	20	–π/6	0.065

**Table 2:** Specific descriptions of comparison methods.

Method Number	Method Name	Principle of MCs Selection
1	DICI Guided PIMO-FMD	FFC
2	DICI Guided PIMO-TT-FMD	FFC
3	PIMO-VMD	
4	VMD-FBE [35]	
5	EWT [36]	FFC
6	MED [37]	
7	SGMD [10]	FFC

**Table 3:** Basic parameters of bearings.

Parameter	Number of Balls	Ball Diameter	Contact Angle	Bearing Mean Diameter
Value	8	7.92 mm	0°	34.55 mm

## Data Availability

All relevant data are included in the paper.
